# Retrospective analysis of linear accelerator output constancy checks using process control techniques

**DOI:** 10.1120/jacmp.v14i1.4032

**Published:** 2013-01-07

**Authors:** Taweap Sanghangthum, Sivalee Suriyapee, Somyot Srisatit, Todd Pawlicki

**Affiliations:** ^1^ Department of Nuclear Technology Chulalongkorn University Bangkok Thailand; ^2^ Division of Radiation Oncology King Chulalongkorn Memorial Hospital Bangkok Thailand; ^3^ Department of Radiation Medicine and Applied Sciences University of California San Diego USA

**Keywords:** output constancy, control chart, process capability index

## Abstract

Shewhart control charts have previously been suggested as a process control tool for use in routine linear accelerator (linac) output verifications. However, a comprehensive approach to process control has not been investigated for linac output verifications. The purpose of this work is to investigate a comprehensive process control approach to linac output constancy quality assurance (QA). The RBA‐3 dose constancy check was used to verify outputs of photon beams and electron beams delivered by a Varian Clinac 21EX linac. The data were collected during 2009 to 2010. Shewhart‐type control charts, exponentially weighted moving average (EWMA) charts, and capability indices were applied to these processes. The Shewhart‐type individuals chart (X‐chart) was used and the number of data points used to calculate the control limits was varied. The parameters tested for the EWMA charts (smoothing parameter (λ) and the control limit width (L)) were λ=0.05, L=2.492; λ=0.10, L=2.703; and λ=0.20, L=2.860, as well as the number of points used to estimate the initial process mean and variation. Lastly, the number of in‐control data points used to determine process capability ($C_{p}$) and acceptability ($C_{\text{pk}}$) were investigated, comparing the first in‐control run to the longest in‐control run of the process data. $C_{p}$ and $C_{\text{pk}}$ values greater than 1.0 were considered acceptable. The 95% confidence intervals were reported. The X‐charts detected systematic errors (e.g., device setup errors). In‐control run lengths on the X‐charts varied from 5 to 30 output measurements (about one to seven months). EWMA charts showed in‐control runs ranging from 9 to 33 output measurements (about two to eight months). The $C_{p}$ and $C_{\text{pk}}$ ratios are higher than 1.0 for all energies, except 12 and 20 MeV. However, 10 MV and 6, 9, and 16 MeV were in question when considering the 95% confidence limits. The X‐chart should be calculated using 8–12 data points. For EWMA chart, using 4 data points is sufficient to calculate the initial mean and variance of the process. The EWMA limits should be calculated with λ=0.10, L=2.703. At least 25–30 in‐control data points should be used to calculate the $C_{p}$ and $C_{\text{pk}}$ indices.

PACS number: 89

## I. INTRODUCTION

Quality assurance (QA) is the process of verifying whether a product or machine function is within some criteria. One purpose of quality assurance in radiotherapy is to ensure constancy of equipment function. Several publications have described QA tools new to radiotherapy; for example: fault tree analysis,^(^ ^1^ ^)^ failure mode and effect analysis,^(^ ^2^ ^)^ or statistical process control (SPC).^(^ ^3^ ^)^ Within SPC, the control chart is a statistical tool used to determine process stability over time and to improve process performance by reducing process variation. Control charts have been used in industrial manufacturing for many years and have also been used in healthcare.^(^ ^4^ ^–^ ^7^ ^)^ More recently, control charts have been applied to radiotherapy quality assurance.^(^ ^3^ ^,^ ^8^ ^–^ ^14^ ^)^

Linear accelerator (linac) output constancy has always been an important part of a regular QA program because the absolute dose delivered to the patient is a major factor in determining the outcome of treatment. While previous reports have indicated the use of control charts for linac output constancy checks,^(^ ^3^ ^)^ no report exists that investigates a comprehensive process control approach to linac output constancy QA. In this study, we apply Shewhart‐type control charts, exponentially weighted moving average (EWMA) charts, and capability indices for linac constancy QA. Our goal is to determine an optimal implementation of these process control tools as part of a comprehensive QA strategy for linac output constancy verification and monitoring.

## II. MATERIALS AND METHODS

The output of a Varian Clinac 21EX linear accelerator machine (Varian Medical Systems, Palo Alto, CA) for 6 and 10 MV photon beams, and 6, 9, 12, 16, and 20 MeV electron beams was calibrated following the IAEA TRS‐398 protocol.^(^ ^15^ ^)^ The routine output verification of all energies were undertaken with a Protea System Corporation Radiation Beam Analyser (RBA‐3) dose constancy check (GAMMEX RMI Inc., Middleton, WI).^(^ ^16^ ^)^ The RBA‐3 consists of five parallel plate chambers of $0.2\;{\text{cm}}^{3}$ volume; one is placed centrally and four other chambers are located on the radial and lateral planes at 8 cm displaced from center. The chambers are covered with the 14 mm lateral Perspex surrounding the chamber, and are placed below a 4 mm thick Perspex sheet. The central ionization chamber reading was used to represent linac output. The RBA‐3 has a thermometer and barometer inside that can correct the chamber signal automatically for temperature and pressure. The data were collected by a physicist or physicist student once per week from January 2009 to August 2010. Fifty monitor units were delivered per reading with a $20\times 20\;{\text{cm}}^{2}$ field size/cone size at 100 cm source‐to‐surface distance (SSD) with 1.8 cm additional buildup of Perspex for 10 MV photon beam and with 0.8 cm additional buildup for all others energies. The RBA‐3 was set up using the optical distance indicator and positioning lasers. We measured the electron mode first, starting from 6, 9, 12, 16, and 20 MeV. When the electron mode was set, 0.8 cm additional buildup was added with applicator size of $20\times 20\;{\text{cm}}^{2}$. For the photon mode, 6 MV used the same 0.8 cm additional buildup as for the electron mode, but used the 1.8 cm additional buildup for 10 MV with $20\times 20\;{\text{cm}}^{2}$ field size. The action limits for all energies was ±3% of baseline.

Following our institutional protocol, at the end of each year, a full calibration of output in a water phantom was done. If the output for any energy was determined to be outside ±1.0% of 1.0 cGy/MU, then the output for that energy was adjusted until the output equalled 1.0 cGy/MU and new baseline values for the RBA‐3 were acquired.

### A. Shewhart‐type control chart

Shewhart control charts consist of an upper control limit (UCL), center line (CL), and a lower control limit (LCL). Data are plotted as a function of the time on the chart. Whenever possible, the data should be partitioned into subgroups. However, control charts are still applicable to individual values. An important assumption when using control charts is that the measurement subgroups (or individual values) are independent. The UCL and LCL are calculated using the linac output data stream and are different for each energy. When the data fall within the UCL and LCL, then the process is said to be in control, and only common (random) causes affect the process. However, if any data point is out of the control limits, then special (nonrandom) causes are affecting the process, and the source(s) of the special cause need to be identified and removed from the process to bring the process back in control. Conventionally, the UCL and LCL are set at ±3 standard deviations from the center line. This implies that 99.7% of the data points would fall within the control limits when the data are normally distributed. Then, when the process is in control, there is only a 0.3% chance that a point will be outside the control limits (i.e., a false positive).

There are two main types of Shewhart control charts that depend on data type: attribute control charts and variable control charts.^(^ ^17^ ^)^ The variable charts are suitable for linear accelerator constancy checks where the data are continuous as opposed to discrete. For the variable control chart, there are individual/moving range (X/MR), average/range ($\bar{X}/R$), and average/standard deviation ($\bar{X}/S$) charts that depend on subgroup size. If the number in each subgroup is less than ten, then the X/MR charts are used.^(^ ^18^ ^)^ If the subgroup size is ten or more, then the $\bar{X}/S$ charts are typically used. Since each output constancy check can be considered a subgroup of size one, individual (X) charts were used. The average and limits are calculated from Eqs. (1) to (3):

(1) $$UCL=\bar{X}+3\frac{\bar{MR}}{d_{2}\sqrt{n}}$$

(2) $$CL=\bar{X}$$

(3) $$LCL=\bar{X}-3\frac{\bar{MR}}{d_{2}\sqrt{n}}$$

where *R* is the range of a subgroup and $d_{2}$ is a bias correction constant that depends on the subgroup size *n.* It is customary to use the constant value $d_{2}=1.128$ for subgroup size n=1 (for a discussion of this, see Pawlicki et al.^(^ ^3^ ^)^ and the references therein). In the case of n=1, the range is taken as the moving range, MR, which is the absolute value of the difference between two consecutive data points $\left({\text{MR}}_{i}=\left|X_{i}-X_{i-1}\right|\right)$. The $\bar{X}$ is calculated as the average over a specified number of data points or subgroups, and the average moving range, $\bar{MR}$, is calculated over the same data points.

We investigated control chart limits as a function of the number of data points to calculate the limits (n=1). The data of first 4, 8, 12, 16, and 20 points, representing to one, two, three, four, and five months, were varied to calculate the control limits in each year for each beam energy. However, if there were any points in the calculation limit that were out of control and the source of the error was known, then those out‐of‐control points were removed and the control limits were recalculated.^(^ ^17^ ^)^ The effect that the number of data used to calculate the control limits has on the detection of out‐of‐control process behavior is investigated by comparing the signal to noise ratio $\left(\bar{x}/\sigma \right)$ over the data used to calculate the limits normalized to the number of data points that were determined to be in control based on those limits.

### B. Exponentially weighted moving average (EWMA) chart

Where the X‐chart is used to detect large changes in the process, the EWMA chart is used to detect gradual drifts in the process. In the EWMA chart, the most recent data points are given a greater weight (λ). The degree of weight reduces with exponential function for prior data with $\lambda \cdot (1-\lambda ),\lambda \cdot {\left(1-\lambda \right)}^{2}$,…, etc. The EWMA equation is given by Eq. (4)

(4) $$EWMA_{t}=\lambda \cdot x_{t}+(1-\lambda )\cdot {EWMA}_{t-1}$$

for t=1, 2, 3, where x is the observation data at time t.

The average and limits for the EWMA chart are calculated from Eqs. (5) to (7):

(5) $$UCL=\mu_{0}+L\cdot \sigma \sqrt{\frac{\lambda }{2-\lambda }[1-{\left(1-\lambda \right)}^{2t}]}$$

(6) $$CL=\mu_{0}$$

(7) $$UCL=\mu_{0}-L\cdot \sigma \sqrt{\frac{\lambda }{2-\lambda }[1-{\left(1-\lambda \right)}^{2t}]}$$

where $\mu_{0}$ is the process average, *t* is the sample number, λ is the weighting factor with values 0<λ≤1, which makes the EWMA chart sensitive to small process drifts. A large λ value means a greater weight to recent data. The parameter L is the width of the control limit. Unlike X‐charts, the UCL and LCL are calculated with each new data point for EWMA charts. However, similar to X‐charts, the EWMA charts also rely on the assumption that the samples (or individual values) are independent.

We varied the parameters λ and L such that λ=0.05, L=2.492; λ=0.10, L=2.703; and λ=0.20, L=2.860, and the number of points used to estimate $\mu_{0}$ and σ were varied from one to five months. Note, $\mu_{0}$ is the same as the $\bar{X}$ in the X‐charts. The values of λ and L were chosen as a range of values that are a compromise between efficient detection of process drifts and chart insensitivity to non‐normal data.^(^ ^17^ ^)^

### C. Process capability and acceptability

For routine linear accelerator output checks, the action limits are ±3.0% of baseline for daily linear accelerator output checks from the AAPM Task Group No.142.^(^ ^19^ ^)^ In this work, we use this same criterion for weekly linear accelerator output checks. The process indices, C and $C_{\text{pk}}$, were chosen for this work because they are industry standards.

**Figure 1:**
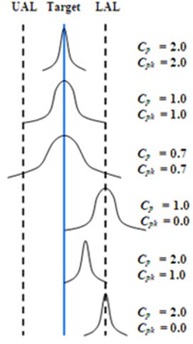
Distributions with different capability ratio ($C_{p}$) and acceptability ratio ($C_{\text{pk}}$) showing their relationship to the process target, upper action limit (UAL), and lower action limit (LAL).

The process capability is used to compare the variation process of the data with respect to the upper and lower action limits to quantify action limit width relative to the dispersion of process data, as shown in Eq. (8):

(8) $$C_{P}=\frac{UAL-LAL}{6\cdot \sigma }$$

where *UAL* and *LAL* are the upper and lower action limits, respectively. The standard deviation of the data distribution is given by σ. The greater the $C_{p}$ value, the better the process is able to meet the action limits, as shown in Fig. 1. A $C_{p}$ value of 1.0 means that the data spread is equal to the action limit width. However, in some processes, a process can still be functioning poorly even with a high $C_{p}$ value (see bottom image in Fig. 1). So, the capability ratio alone is not enough to provide a full description of a process because $C_{p}$ does not indicate the degree to which the process is centered on the target.

The process acceptability is another index that should be used to fully characterize a process. Acceptability describes how close the process center is to the nearest action limit. It is calculated from Eq. (9):

(9) $$C_{pk}=\min(\frac{UAL-\mu }{3\cdot \sigma },\frac{\mu -LAL}{3\cdot \sigma })$$

If the process is on target, the value of the capability ratio will be equal to the acceptability ratio. However, the capability ratio is higher than acceptability ratio in cases where a process is not on target, as shown in Fig. 1. When calculating $C_{p}$ and $C_{\text{pk}}$ ratios, it is important that the process is in control, that is, no points are outside the control limits on the X‐chart. The reason for this requirement is if the process is changing, then one cannot be confident that the process is subject to only random (or common) causes. Furthermore, the normal distribution upon which the capability and acceptability ratios depend is not assured. In this study, data normality was verified using the Anderson‐Darling test statistic. For any non‐normal distributions, the Johnson Transformation was used to normalize the data. The normalized data were then used to calculate $C_{p}$ and $C_{\text{pk}}$. The Anderson‐Darling tests and Johnson Transformations were done in Minitab v16 software (Minitab Inc, State College, PA).

To investigate the impact of run length on the interpretation of capability and acceptability, we have used the first run and longest run of in control points in each year to calculate the $C_{p}$ and $C_{\text{pk}}$ for each energy, as well as the number of data points used to calculate the X‐chart limits upon which the $C_{p}$ and $C_{\text{pk}}$ are determined. The first run is the number of data points until reaching the first out‐of‐control point, while the longest run is the number of longest consecutive data points within the control limits.

It is important to note that $C_{p}$ and $C_{\text{pk}}$ in Eqs. (8) and (9) are usually point estimates approximated by using the sample standard deviation (or $MR/d_{2}$) to estimate σ and the sample average to estimate μ. Therefore, $C_{p}$ and $C_{\text{pk}}$ are subject to statistical fluctuations and confidence intervals should be reported. We used the sample standard deviation and average to determine $C_{p}$ and $C_{\text{pk}}$ and results are reported at the 95% confidence interval.

## III. RESULTS

### A. Shewhart‐type chart

**Figure 2:**
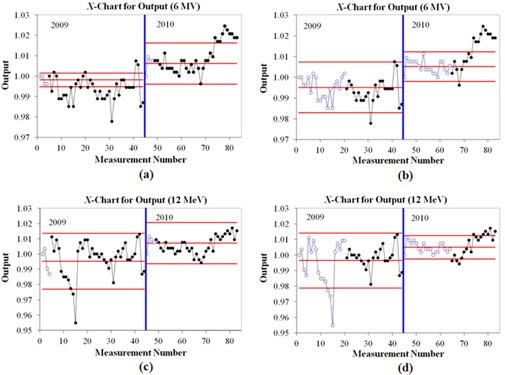
The X‐control chart for output constancy check for 6 MV (a) and (b) and 12 MeV (c) and (d). The output data in first month used to calculate the control limit are displayed in (a) and (c), while (b) and (d) used four months of data. The solid lines are the process behavior limits and the center line. The open circles are the data points used to calculate the control limits. Point 1 corresponds to the date 12/10/2008, point 45 to 11/10/2009, and point 83 to 8/30/2010. The solid vertical (blue) line separates data collected in 2009 from that collected in 2010.

The X‐charts for output consistency of 6 MV and 12 MeV are shown in Fig. 2. The first 44 data points belong to year 2009, and the remainder are the output values for year 2010. The two figures on the left use first month of data to calculate control limits, while right two figures use first four months for calculating limits.

In 2009, point number 15 was a systematic error owing to setup error where a junior physicist set a 100 cm SSD on surface of RBA‐3 instead of a 100 cm SSD on Perspex phantom. For all charts where point 15 was out of control over the time used to calculate the limits, it was removed and new control limits were calculated. This resulted in only an average of 0.1% change in the control chart limit width for all energies. The average output in year 2009 was lower than 1.0 cGy/MU, while most of the output data in year 2010 were higher than 1.0 cGy/MU. Point number 31 also had an error in RBA‐3 setup. The outputs were checked the day after (point 32). When using one month of data to calculate the limits, point number 43 was out of control for almost all energies (but not for 12 MeV, as shown in Fig. 2). The measurements were repeated two days later (point 44) and the result still showed out‐of‐control process behavior (e.g., Fig. 2(a). Therefore, it was decided to do the full physics calibration in water phantom, which confirmed deviations of more than 1.0 cGy/MU for all energies. The outputs were then calibrated to 1.0 cGy/MU starting at point 45 (Fig. 2(a)‐(d)). After point 74 in 2010, the process showed consistent out‐of‐control behavior. A full calibration was done about one month before the scheduled time (i.e., at point 84).

The number of consecutive in‐control data points on X‐charts is important because in‐control process behavior is the basis for the correct interpretation of process capability and acceptability. Table 1 displays the number of first run points and number of longest run points before an out‐of‐control point is detected from the X‐chart for 6 and 10 MV photon beams, and 6, 9, 12, 16, and 20 MeV electron beams with different number of data points used to calculate the limits.

**Table 1:** The number of first run points before out‐of‐control limits and number of longest run points on the X‐charts for 6 and 10 MV photon beams, and 6, 9, 12, 16, and 20 MeV electron beams. Each month is equal to 4 data points. N is the number of data points used to calculate the limits.

*N*	*6X '09*		*10X '09*		*6E '09*		*9E '09*		*12E '09*		*16E '09*		*20E '09*	
*(months)*	$1^{\text{st}}$	*Long*	$1^{\text{st}}$	*Long*	$1^{\text{st}}$	*Long*	$1^{\text{st}}$	*Long*	$1^{\text{st}}$	*Long*	$1^{\text{st}}$	*Long*	$1^{\text{st}}$	*Long*
1	5	5	5	7	5	5	8	11	13	29	6	12	5	11
2	12	15	12	15	14	15	14	15	13	29	14	15	14	15
3	12	15	14	15	14	15	14	15	13	29	14	15	14	15
4	30	30	30	30	30	30	14	15	13	29	14	15	14	29
5	30	30	30	30	14	15	12	15	12	29	14	15	14	15

**Figure 3:**
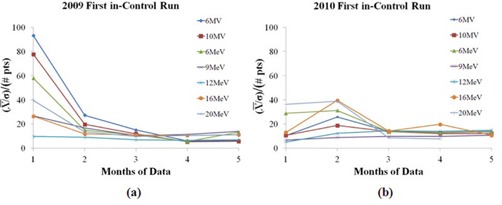
Signal to noise ratio ($\left(\bar{x}/\sigma \right)$) normalized by the number of in‐control data points for the data in (a) 2009 and (b) 2010 for the first run of the data in control for all energies.

Figure 3 shows signal to noise ratio for each energy in 2009 and 2010. There is a clear trend that by month two or three (8 to 12 data points), the limits are stable. The process is more stable in 2010 (see Fig. 3(b), which shows that the overall value of normalized signal to noise is improved.

### B. Exponentially weighted moving average (EWMA) chart

**Figure 4:**
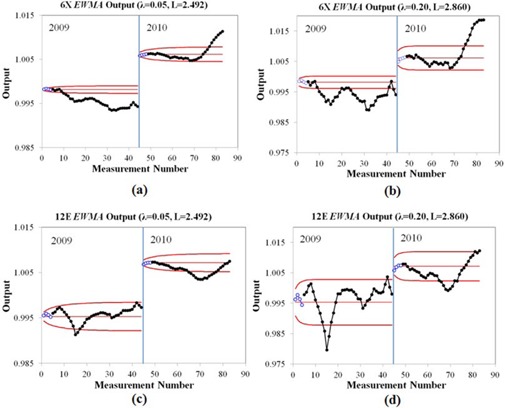
The exponentially weighted moving average (EWMA) chart for output constancy check measured by central ionization chamber of RBA‐3 device with first month calculated control limits for (a) 6 MV, λ=0.05, L=2.492; (b) 6 MV, λ=0.20, L=2.860; (c) 12 MeV, λ=0.05, L=2.492; and (d) 12 MeV, λ=0.20, L=2.860. The open circles are the points used to calculate control limit and the filled dots are collecting output data. The solid red line represents control limits.

EWMA charts are useful to detect slow drifts of a process. The EWMA chart in Fig. 4 displays the output measurements for 6 MV photon beam and 12 MeV electron beam with λ=0.05, L=2.492, and λ=0.20, L=2.860, and using one month (4 points) in the calculation to estimate $\mu_{0}$ and σ. The greater λ and L are selected, the larger limit width becomes, as shown in Fig. 4. The processes for all energies exhibit out‐of‐control behavior. For 12 MeV in 2009 and different λ and L, the EWMA charts detect out‐of‐control process behavior at points 14 or 15. Similar results are found for 6 MV in 2009 at point 9 or 10. For 6 MV in 2009 (λ=0.2), the process wanders in and out of control starting at point 10 and similar behavior is seen for the 12 MeV process.

Table 2 shows the number of first in‐control run of points from the EWMA chart for photon beams and some of the electron beams. For the results in this table, one to three months (4–12 points) were used to calculate the control limits for different λ and L parameters. For a given number of points used to estimate $\mu_{0}$ and σ, there were not a significantly different number of first run points in control for different λ and L parameters (Table 2). The processes are stable over a longer period of time in 2010 compared to 2009 before eventually going and remaining out of control.

**Table 2:** The number of measurements before the first out‐of‐control point is observed on the EWMA charts for 6 and 10 MV photon beams, and 6, 12, and 20 MeV electron beams using one to three months (4–12 data points) to calculate the control limits for different smoothing parameters (λ) and limit widths (L). N is the number of data points used to calculate the limits.

*N*	*Parameters*	*6X*		*10 X*		*6E*		*12E*		*20E*	
*(months)*		*′09*	*′10*	*′09*	*′10*	*′09*	*′10*	*′09*	*′10*	*′09*	*′10*
1	λ=0.05 L=2.492	9	33	8	24	9	13	15	21	13	13
	λ=0.10 L=2.703	10	32	9	24	9	13	15	21	12	12
	λ=0.20 L=2.860	10	31	9	24	9	13	14	21	12	11
2	λ=0.05 L=2.492	11	24	12	23	12	13	13	21	12	11
	λ=0.10 L=2.703	11	24	11	23	12	13	12	21	12	11
	λ=0.20 L=2.860	10	24	11	24	10	12	12	21	12	11
3	λ=0.05 L=2.492	31	24	29	24	29	21	15	22	0	17
	λ=0.10 L=2.703	31	24	16	24	29	21	15	21	15	16
	λ=0.20 L=2.860	15	24	15	24	15	21	14	21	15	15

### C. Process capability and acceptability

**Figure 5:**
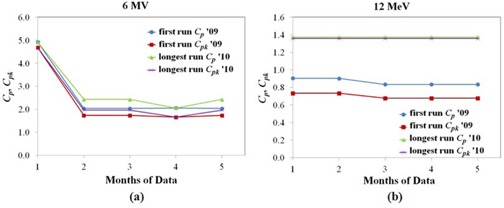
The capability ratio ($C_{p}$) and acceptability ratio ($C_{\text{pk}}$) of first run and longest run for output constancy check measured by central ionization chamber of RBA‐3 device with different time to calculate the control limits for (a) 6 MV photon beams and (b) 12 MeV electron beams.

Process capability ratio and process acceptability ratio, $C_{p}$ and $C_{\text{pk}}$, were used to characterize the process performance of radiation routine output the linac. The data for the year 12 MeV in 2009 and 6 MV, 9 MeV, and 20 MeV in 2010 were nonnormal and consequently transformed to normal prior to calculating the process capability and acceptability. Figure 5 displays the $C_{p}$ and $C_{\text{pk}}$ values with different times to calculate the control limit for 6 MV (a) and 12 MeV (b) in both first run and longest run for 2009 and 2010.

**Figure 6:**
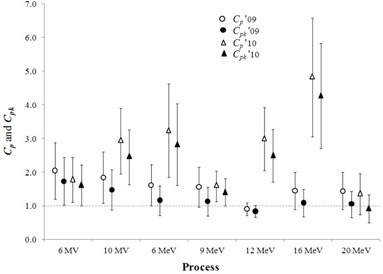
The capability ratio ($C_{p}$) and acceptability ratio ($C_{\text{pk}}$) with the 95% confidence interval for the process of linac output verification per energy using the first in‐control run and two months of data to calculate the control limits. Values of $C_{p}$ and $C_{\text{pk}}$ above the dashed horizontal line are considered acceptable (also see Fig 1).

Figure 6 shows a comparison of calculated $C_{p}$ and $C_{\text{pk}}$ with 95% confidence interval calculated using the first in‐control run of data for all photon and electron energies in the years 2009 and 2010. The result showed $C_{\text{pk}}$ values were lower than $C_{p}$ values for all energies, which implied the process has some shift from the target values and is also evident on the X‐charts. However, both $C_{p}$ and $C_{\text{pk}}$ were higher than 1.0 for most energies except 12 MeV in year 2009 and 20 MeV in 2010.

## IV. DISCUSSION

The output data should be compared to clinically appropriate action limits to decide whether or not a specific data point is acceptable for clinical use at that instant in time.^(^ ^19^ ^)^ For process monitoring and improvement, the output data needs to be compared to the control limits. Our data indicate that the control limits are typically smaller than the action limits for routine output verification. The goal, then, is to calculate the control limits as soon as reliable limits are achievable. The results in Fig. 2 and Table 1 demonstrate that using only four points (one month) to calculate the control limits results in variable limits. Note that in Fig. 2, point 15 is clearly out of control for the 12 MeV, but is not correspondingly out of control for 6 M V. The reason for this is when the junior physicist finished the measurements in electron mode, she entered the room to remove the applicator for the photon measurements. She subsequently checked the SSD at that time and found and corrected the setup error before the photon measurements. It is important to keep the in‐error data points on the chart because it provides information about the process. A retrospective analysis can also be performed to investigate the stability of the linear accelerator/measurement system. This retrospective analysis would only omit data points that were investigated and known to be resultant from errors that have been remediated (e.g., device setup errors that are controlled by starting a new training program). When the number of data points to calculate the limits was increased, the results become more consistent. This is demonstrated in the normalized signal to noise ratio of Fig. 3. This figure also shows that a more stable process (e.g., data of 2010) leads to better normalized signal to noise ratio (lower values in 2010 compared to 2009), which is reflected in the longer runs of in‐control points. However, as an issue of process control, there is often very little one can do to the process in the clinic routine to increase the signal to noise ratio. On the other hand, one method to achieve this is to create larger groups of your time‐ordered data. This is the intent of Fig. 3, namely that even if the process is performing erratically as observed in 2009 compared to 2010 (see Fig. 2), one can group data points, thereby minimizing the effects of noise. This is shown in the Fig. 3 where four or five months of data are used, the signal to noise ratio is roughly the same in 2009 and 2010. Based on these results, we recommend that between two to three months of data (8–12 data points) should be used to calculate the control limits. This is also consistent with the findings of Pawlicki et al.^(^ ^11^ ^)^ for data from IMRT QA point dose measurements compared to planning systems or independent computer verifications. It should be reiterated that each data point is also compared to the clinical action limits for acceptability. There is no risk to the patient in using one month (4 data points), for example, to calculate the control limits. However, unstable control limits means that one may miss some process changes if the limits are too wide, or experience some false positives if the limits are too narrow. One can mitigate anomalous interpretation of process behavior by calculating the control limits after 8–12 data points have been acquired.

If out‐of‐control points occurred over the time used to calculate the control limits and the reason for those out‐of‐control points is known (and can be remediated), then those out‐of‐control points could be removed from the control limit calculations. However, based on our results, change in the control chart limit width is small when removing these points. Therefore, one can omit this procedure when calculating control limits without affecting the usefulness of the charts. It is not necessary to be overly concerned with being overly precise in determination of the control limits. It is more important to use the correct procedure to calculate the limits, and that control limits should be calculated for each energy and each machine. Analysis of output constancy using this approach will tell more about the process than using a one‐size‐fits‐all action limit approach to output constancy verification.

Control limits are point binomial estimates and there is an uncertainty associated with the calculation (similar to the process capability and acceptability indices). Determining confidence limits on each control limit is again overly complicating the procedure and would likely make interpretation of the results more complicated. For process capability and acceptability, it makes sense to calculate confidence limits because those ratios are used to make a definitive statement of process performance at a specific instance in time.

Gerard et al.^(^ ^12^ ^)^ presented the use of EWMA charts for IMRT QA. It was concluded that EWMA charts were an efficient tool to detect the small and slow drifts occurred from MLC error in their IMRT dose delivery process. However, effects of the smoothing parameter (A.) and the control limit width (L) were not presented. Our investigation of different values of A, and L indicate that when the parameters of λ and L increase, the limit width is also larger. Figure 4 and Table 2 demonstrate that using 4 data points (one month) results in initial parameters $\mu_{0}$, σ, and control limits that efficiently detected the slow process changes. We surmised that the slow process change was due to linac ouptut drifts due to the linac monitor ion chamber, as described by Grattan and Hounsell.^(^ ^20^ ^)^ The electron energies of 9 MeV and 16 MeV are slightly less stable than the rest of the energies. Since some of beam control components are independently controlled for each energy and modality (e.g., independent control boards within the linac control cabinet), we attribute the differences noted in Table 2 to the energies being able to drift independently. As to the choice of λ and L, we recommend that λ=0.1 and L=2.703 be used. This choice is based on the fact that the EWMA control limits are narrower than the ±3.0% action limits. If the data had a very large drift variability with EWMA control limits greater than ±3.0%, it might be advisable to use λ=0.05 and L=2.492 to quickly identify and correct the reason for the drifting process. Almost all of the out‐of‐control points on the X‐chart were due to RBA‐3 setup errors from junior physicist or physics student. This indicates that efforts toward more training and/or standardization are warranted. The slow linac drift errors were detected on the EWMA charts and eventually on the X‐chart, as well.

Although the process for all energies gradually changed after point 74, the process was still within the clinical action limits. Because the EWMA chart is not as effective in detecting sudden large shifts in the process and the X‐chart is relatively slow in responding to gradual shift process shifts, using these two charts together might be the best approach for online process monitoring, as indicated by Woodall and Mahmoud.^(^ ^21^ ^)^ However, there is some indication that EWMA charts can be used without X‐charts to detect both large process changes and slow drifts, so long as the EWMA charts are based on the squared deviations from the target.^(^ ^22^ ^)^ This could be a direction for future investigations.

Gerard et al.^(^ ^12^ ^)^ simultaneously evaluated long‐term capability indices ($P_{p}$,$P_{\text{pk}}$, and $P_{\text{pm}}$) to the process of IMRT QA. Long‐term means these indices are applied over long runs of a process when a process may or may not be in control so one needs to be careful when interpreting long‐term capability indices. Breen et al.^(^ ^10^ ^)^ and Nordström et al.^(^ ^14^ ^)^ applied $C_{p}$ and $C_{\text{pk}}$ to the processes of IMRT QA and independent computer calculation checks, respectively. Both authors make a distinction when calculating $C_{\text{pk}}$ for nonnormal data by using the nonparametric form of those equations. In this work, we use the parametric versions of the indices, but transformed the nonnormal distributions. The previous works did not report confidence intervals, which we feel are important to understanding the reliability of the process ability and capability. If we consider the 95% confidence interval, then only the 6 MV process is both capable and acceptable in 2009, whereas only the 20 MeV process is neither capable nor acceptable in 2010. When using only a few data points (e.g., ≤25), the point estimates $C_{p}$ and $C_{\text{pk}}$ are associated with a large variability (Fig. 6 and Table 1). Given these issues, we recommended waiting to calculate the $C_{p}$ and $C_{\text{pk}}$ until there are at least 25 or more in‐control data points. Even though we used the sample standard deviation to calculate $C_{p}$ and $C_{k}$, if the data are in control and follow a normal distribution, then the same results are obtained when the estimate $MR/d_{2}$ is used to calculate $C_{p}$ and $C_{\text{pk}}$. Ultimately, other process indices such as $C_{\text{pc}}$ ^(^ ^23^ ^)^ or $C_{\text{pm}}$ ^(^ ^24^ ^)^ that are insensitive to the form of the distribution and simultaneously evalute the process variability and centering may be better. In any case, process indices should be used for high‐level communication or documentation about process performance — for example, to department administrators, accreditation bodies, or inter‐institutional process comparisons.

In this work, linac output constancy was verified on a weekly basis. There is evidence in the literature that one learns similar things about the process of routine output checks from a frequency of daily checks.^(^ ^3^ ^)^ However, continued use of the charts in daily and weekly frequency schemes could highlight issues that have heretofore gone unappreciated. The frequency of any QA activity depends on factors such as magnitude of error that could result if not checked, time and cost of the QA procedures, and the opportunity costs of not being able to do other work. Due to the possible errors if the linac output deviates significantly from the baseline, output verification should be performed daily. The results of this work are also applicable to daily output checks. In the case of daily output checks, one can use the first two to three weeks of data to construct the X‐chart and the first week of data to build the EWMA chart.

Lastly, one should take care not to adjust the process within the noise of the system. If the control charts still show output is constant within the control limits (and within clinical action limits), then the full calibration in water phantom would still be performed, but output adjustment might be not necessary. Optimal strategies for process adjustments should be considered, but this is out of the scope of this work and an area for future research.

## V. CONCLUSIONS

The conclusions from this investigation are the following: 1) The concept of industrial engineering QA using X‐charts, EWMA charts, and process capability and acceptability indices provide a new perspective on the process of routine linac output verifications; 2) the first 8–12 data points (two to three months for weekly verifications and two to three weeks for daily verifications) should be used to calculate X‐chart control limits, the first 4–6 data points (one month for weekly verifications and one week for daily verifications) should be used to calculate EWMA control limits using λ=0.1 and L=2.703; and 3) at least 25–30 in‐control data points should be used to calculate process capability ($C_{p}$) and process acceptability ($C_{\text{pk}}$) using at 95% confidence interval, otherwise a “not able to be reported” should be documented.

## ACKNOWLEDGMENTS

The authors gratefully acknowledge the 90th Anniversary of Chulalongkorn University Fund (Ratchadaphiseksomphot Endowment Fund) and the IAEA's Doctoral Coordinated Research Project on “QA of the Physical Aspects of Advanced Technology in Radiotherapy” (E2.40.15) for financial support.
